# Artificial chameleon skin that controls spectral radiation: Development of Chameleon Cool Coating (C^3^)

**DOI:** 10.1038/s41598-018-19498-5

**Published:** 2018-01-19

**Authors:** Hiroki Gonome, Masashi Nakamura, Junnosuke Okajima, Shigenao Maruyama

**Affiliations:** 1 Department of Mechanical Engineering, Shibaura Institute of Technology, 3-7-5 Toyosu, Koto-ku, Tokyo, 135–8548 Japan; 2 School of Engineering, Tohoku University, 6-6, Aoba, Aramaki-aza, Aoba-ku, Sendai, Miyagi 980–8579 Japan; 3 Institute of Fluid Science, Tohoku University, 2-1-1, Katahira, Aoba-ku, Sendai, Miyagi 980–8577 Japan

## Abstract

Chameleons have a diagnostic thermal protection that enables them to live under various conditions. Our developed special radiative control therefore is inspired by the chameleon thermal protection ability by imitating its two superposed layers as two pigment particles in one coating layer. One particle imitates a chameleon superficial surface for color control (visible light), and another particle imitates a deep surface to reflect solar irradiation, especially in the near-infrared region. Optical modeling allows us to optimally design the particle size and volume fraction. Experimental evaluation shows that the desired spectral reflectance, i.e., low in the VIS region and high in NIR region, can be achieved. Comparison between the measured and calculated reflectances shows that control of the particle size and dispersion/aggregation of particle cloud is important in improving the thermal-protection performance of the coating. Using our developed coating, the interior temperature decreases and the cooling load is reduced while keeping the dark tone of the object.

## Introduction

**Figure 1:**
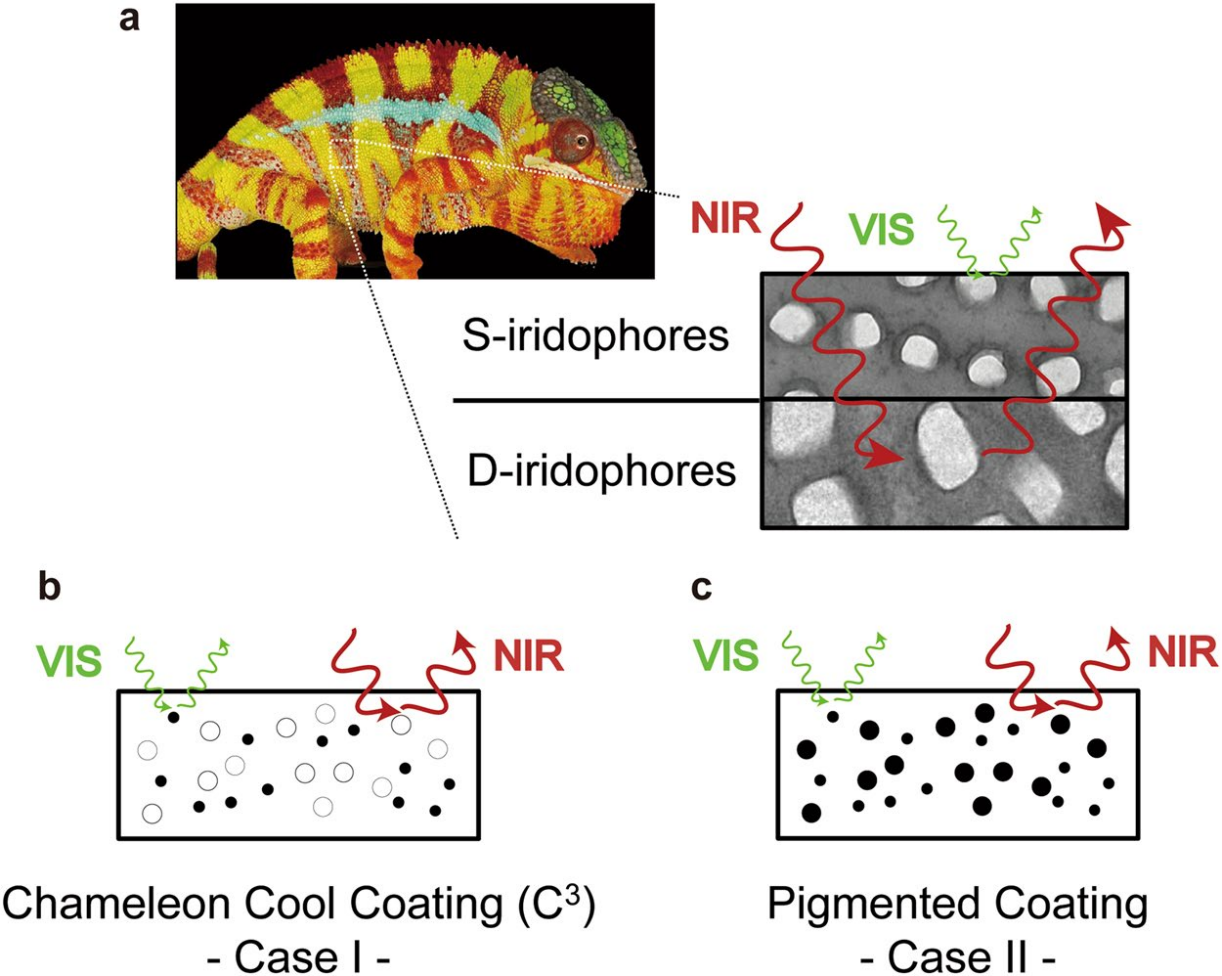
C^3^ Concept. (**a**) Schematic of the thermal barrier in a chameleon. Picture of chameleon and transmission electron microscope image of guanine nanocrystals in superficial (S-) and deep (D-) iridophores obtained from a paper written by Teyssier *et al*.^4^. We acknowledge their excellent work. (**b**) Schematic of C^3^. The black particle controls the VIS light, and the white particle reflects the NIR light. (**c**) Schematic of a usual coating pigmented by a single particle.

Spectral control has been developed as an important technology in thermal engineering. In nature, such complex radiative control has already been realized by *morpho* butterflies^1–3^, chameleons^4^, and other animals^5–10^. A chameleon controls light by dispersion (or aggregation) of pigment-containing organelles within its dermal chromatophores. It organizes iridophores into two superposed layers to create efficient camouflage with spectacular display while potentially providing a passive thermal protection. Within the superficial thick layer of the dermal iridophores, chameleons change color [visible (VIS) light] by active tuning a lattice of guanine nanocrystals. A denser population of iridophores with large crystals reflects a substantial proportion of sunlight, especially in the near-infrared (NIR) range (Fig. 1a).

The radiative control by a chameleon, which controls VIS light and reflects NIR light, is ideal in many cases. For example, products such as cars and buildings^11–14^ are usually exposed to too much sunlight. In many cases, dark-color coatings are preferably used in these products for appearance purposes. However, these dark coatings absorb a large amount of solar irradiation in the VIS and NIR regions. Therefore, the interior temperature becomes high and creates a large cooling load in air-conditioning systems. The resultant energy consumption contributes to environmental problems such as the greenhouse effect^15^ and urban heat islands^16^. In a situation where a surface must be kept cool when exposed to sunlight, the surface should have maximum solar-energy reflectance. We designed a bio-inspired Chameleon Cool Coating (C^3^) by imitating two superposed layers as two pigment particles in one coating layer (Fig. 1b) to simultaneously achieve two contradictory properties: “black” and “cool.” One particle controls the color (VIS light), and the other particle reflects NIR light. Single layer makes us easy to introduce our coating into practical use. We defined the optimization parameter and quantitatively evaluated the spectral selectivity performance. This combination of two functional particles constitutes an evolutionary novelty that allows us to more precisely control the spectrum while protecting against the thermal consequences of intense solar radiations.

## Results

### Effect of particle size on the optimization parameter

**Figure 2:**
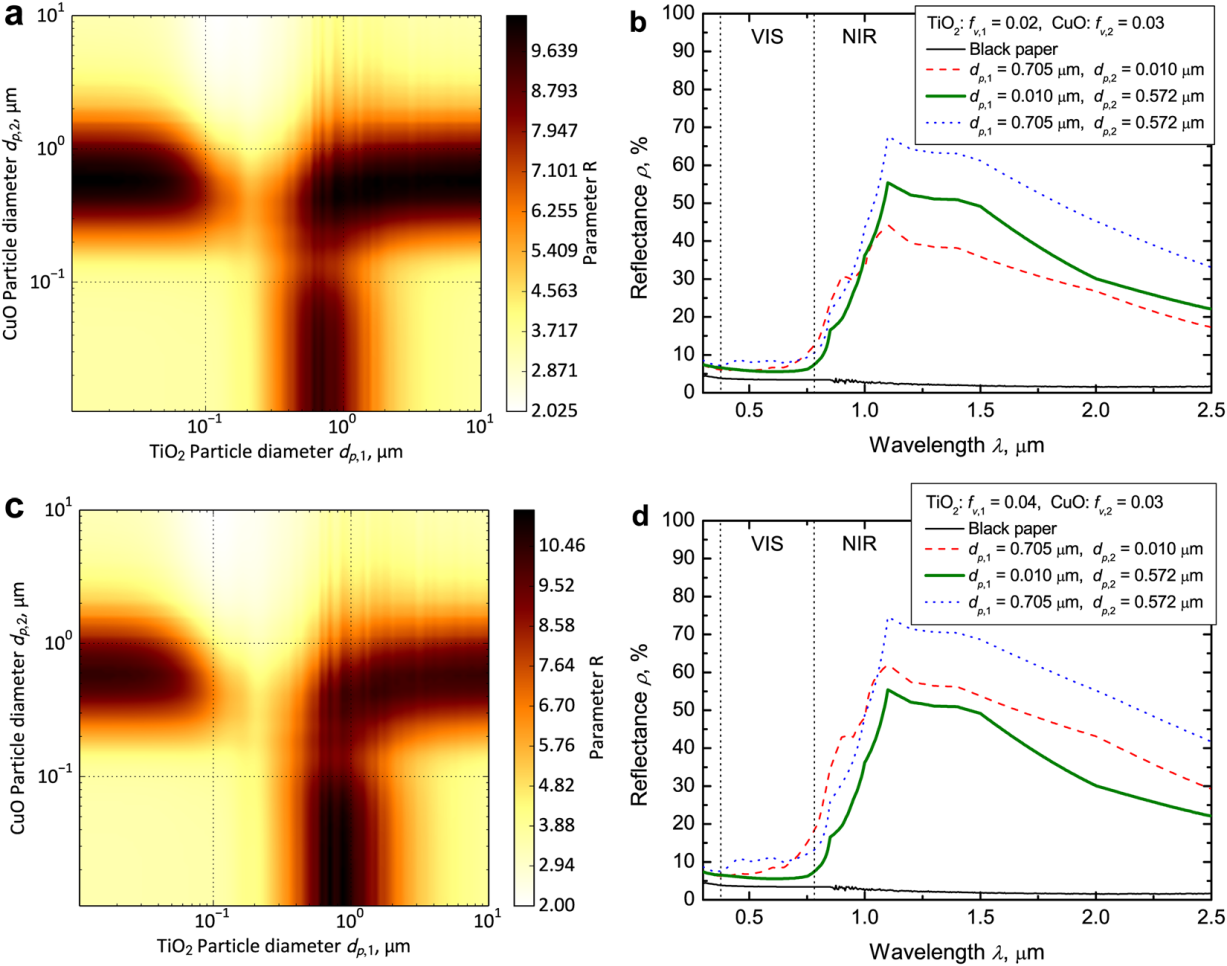
Effect of particle diameter on the optimization parameter when the VIS controlling particle is CuO and the NIR reflecting particle is TiO_2_. (**a**) Color map of *R* when *f*_*v*,1_ is 0.02 and *f*_*v*,2_ is 0.03. Optimization parameter *R* is maximum (*R* = 10.38) when *d*_*p*,1_ is 0.010 μm and *d*_*p*,2_ is 0.572 μm. (**b**) Calculated spectral reflectance of C^3^ when *f*_*v*,1_ is 0.02 and *f*_*v*,2_ is 0.03, *d*_*p*,1_ is 0.705 μm and *d*_*p*,2_ is 0.010 μm (red dashed curve; *R* = 9.11), *d*_*p*,1_ is 0.010 μm and *d*_*p*,2_ is 0.572 μm (green bold curve; *R* = 10.38), *d*_*p*,1_ is 0.705 μm and *d*_*p*,2_ is 0.572 μm (blue dotted curve; *R* = 9.20), and bare black paper (black curve). (**c**) Color map of *R* when *f*_*v*,1_ is 0.04 and *f*_*v*,2_ is 0.03. Optimization parameter *R* is maximum (*R* = 10.59) when *d*_*p*,1_ is 0.705 μm and *d*_*p*,2_ is 0.010 μm. (**d**) Calculated spectral reflectance of C^3^ when *f*_*v*,1_ is 0.04 and *f*_*v*,2_ is 0.03, *d*_*p*,1_ is 0.705 μm and *d*_*p*,2_ is 0.010 μm (red dashed curve; *R* = 10.59), *d*_*p*,1_ is 0.010 μm and *d*_*p*,2_ is 0.572 μm (green bold curve; *R* = 10.37), *d*_*p*,1_ is 0.705 μm and *d*_*p*,2_ is 0.572 μm (blue dotted curve, *R* = 8.34), and bare black paper (black curve).

We calculated the spectral reflectance of C3 and its optimization parameter R. The particle size greatly affects the spectrum. R varies with the TiO_2_ and CuO particle diameters when the volume fractions of the TiO_2_ and CuO particles are constant, where volume fraction of the TiO_2_ particle *f*_*v*,1_ is 0.02 and that of the CuO particle *f*_*v*,2_ is 0.03 (Fig. 2a). R is maximum (R = 10.38) when TiO_2_ particle diameter *d*_*p*,1_ is 0.010 μm and CuO particle diameter *d*_*p*,2_ is 0.572 μm. Under these conditions, the spectral reflectance is low in the VIS region and high in the NIR region (green bold curve in Fig. 2b). On the other hand, R is maximum (R = 10.59) when *d*_*p*,1_ is 0.705 μm and *d*_*p*,2_ is 0.010 μm if *f*_*v*,1_ is 0.04 and *f*_*v*,2_ is 0.03 (Fig. 2c red dashed curve in Fig. 2d). The optimized combination of TiO_2_ and CuO particle sizes differs in terms of the volume fractions of the TiO_2_ and CuO particles. This result indicates that controlling the spectral reflectance is important to control the particle size and volume fraction. When the effective diameters of the TiO_2_ and CuO particle (*d*_*p*,1_ = 0.705 μm and *d*_*p*,2_ = 0.572 μm) are combined, the spectral reflectance is high in the NIR region (blue dotted curves in Fig. 2b,d). However, R is low because C3 has a gray color (high VIS reflectance).

### Effect of volume fraction on the optimization parameter

**Figure 3:**
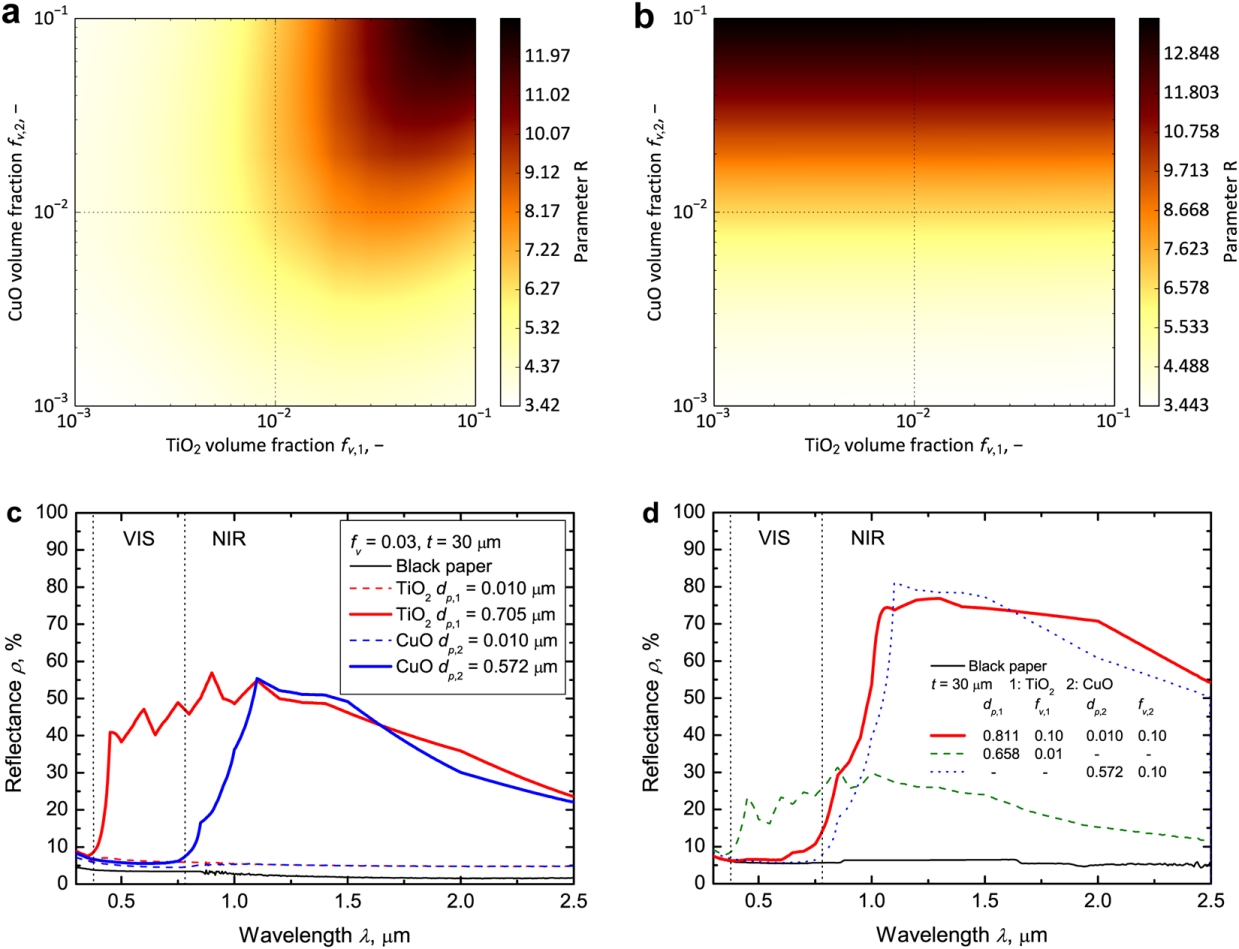
Effect of volume fraction on the optimization parameter when the VIS controlling particle is CuO and the NIR reflecting particle is TiO_2_. (**a**) Color map of *R* when *d*_*p*,1_ is 0.705 μm and *d*_*p*,2_ is 0.010 μm. *R* is maximum (*R* = 12.89) when *f*_*v*,1_ is 0.09 and *f*_*v*,2_ is 0.10. (**b**) Color map of *R* when *d*_*p*,1_ is 0.010 μm and *d*_*p*,2_ is 0.572 μm. *R* is maximum (*R* = 13.76) when *f*_*v*,1_ is 0.001 and *f*_*v*,2_ is 0.10. (**c**) Calculated spectral reflectance of the TiO_2_ pigmented coating when the *d*_*p*,1_ values are 0.010 μm (red dashed curve) and 0.705 μm (red bold curve) and that of the CuO pigmented coating when the *d*_*p*,2_ values are 0.010 μm (blue dashed curve) and 0.572 μm (blue bold curve). (**d**) Calculated spectral reflectance of C^3^ when *d*_*p*,1_ is 0.811 μm, *f*_*v*,1_ is 0.10, *d*_*p*,2_ is 0.010 μm, and *f*_*v*,2_ is 0.10 (red bold curve; *R* = 13.93) and that when *d*_*p*,1_ is 0.010 μm, *f*_*v*,1_ is 0.01 (green dashed curve; *R* = 3.84), *d*_*p*,2_ is 0.572 μm, *f*_*v*,2_ is 0.10 (blue dotted curve; *R* = 13.79), and black paper (black curve).

*R* varies with the volume fractions of the TiO_2_ and CuO particles when the TiO_2_ and CuO particle diameters are constant, where *d*_*p*,1_ is 0.705 μm and *d*_*p*,2_ is 0.010 μm (Fig. 3a). *R* is maximum (*R* = 12.89) when *f*_*v*,1_ is 0.09 and *f*_*v*,2_ is 0.10. In the combination of the TiO_2_ and CuO particle diameters, a higher volume fraction of the TiO_2_ particle is desired to have a high spectral reflectance in the NIR region (red bold curve in Fig. 3c). Simultaneously, a high volume fraction of CuO particle is desired to cancel the high spectral reflectance in the VIS region due to the absorption of the small CuO particle (blue dashed curve shown in Fig. 3c). On the other hand, *R* is maximum (*R* = 13.76) when *f*_*v*,1_ is 0.001 and *f*_*v*,2_ is 0.10 if *d*_*p*,1_ is 0.010 μm and *d*_*p*,2_ is 0.572 μm (Fig. 3b). In the combination of the TiO_2_ and CuO particle diameters, a higher volume fraction of the CuO particle is desired to have high spectral reflectance in the NIR region (blue bold curve in Fig. 3c). The CuO particle can simultaneously maintain low VIS and high NIR reflectances. Additionally, a coating pigmented with a small TiO_2_ particle has only a low VIS reflectance (red dashed curve in Fig. 3c). Therefore, the calculation shows that the best *f*_*v*,1_ value is 0.001 and indicates that no other supporting particle is necessary (Fig. 1c).

### Best combination of particle diameters and volume fractions

*R* is calculated in 7,683,984 (99 × 99 × 28 × 28) combinations, where *d*_*p*,1_ and *d*_*p*,2_ are 0.010 and 10 μm (logarithmically divided into 99 cases), respectively, and *f*_*v*,1_ and *f*_*v*,2_ are 0.001 and 0.1 (logarithmically divided in 28 cases), respectively. *R* is highest (*R* = 13.93) when *d*_*p*,1_ is 0.811 μm, *f*_*v*,1_ is 0.10, particle *d*_*p*,2_ is 0.010 μm, and *f*_*v*,2_ is 0.10 (red bold curve in Fig. 3d). The spectral reflectance of C^3^ reaches 75% in the NIR region with a black color (low VIS reflectance). The spectral selectivity performance is higher than that of the coating pigmented by a single particle. The highest optimization parameters are 3.84 and 13.79 for TiO_2_ and CuO pigment coatings, respectively (green and blue dotted curves in Fig. 3d).

### Measured spectral reflectance of C^3^

**Figure 4:**
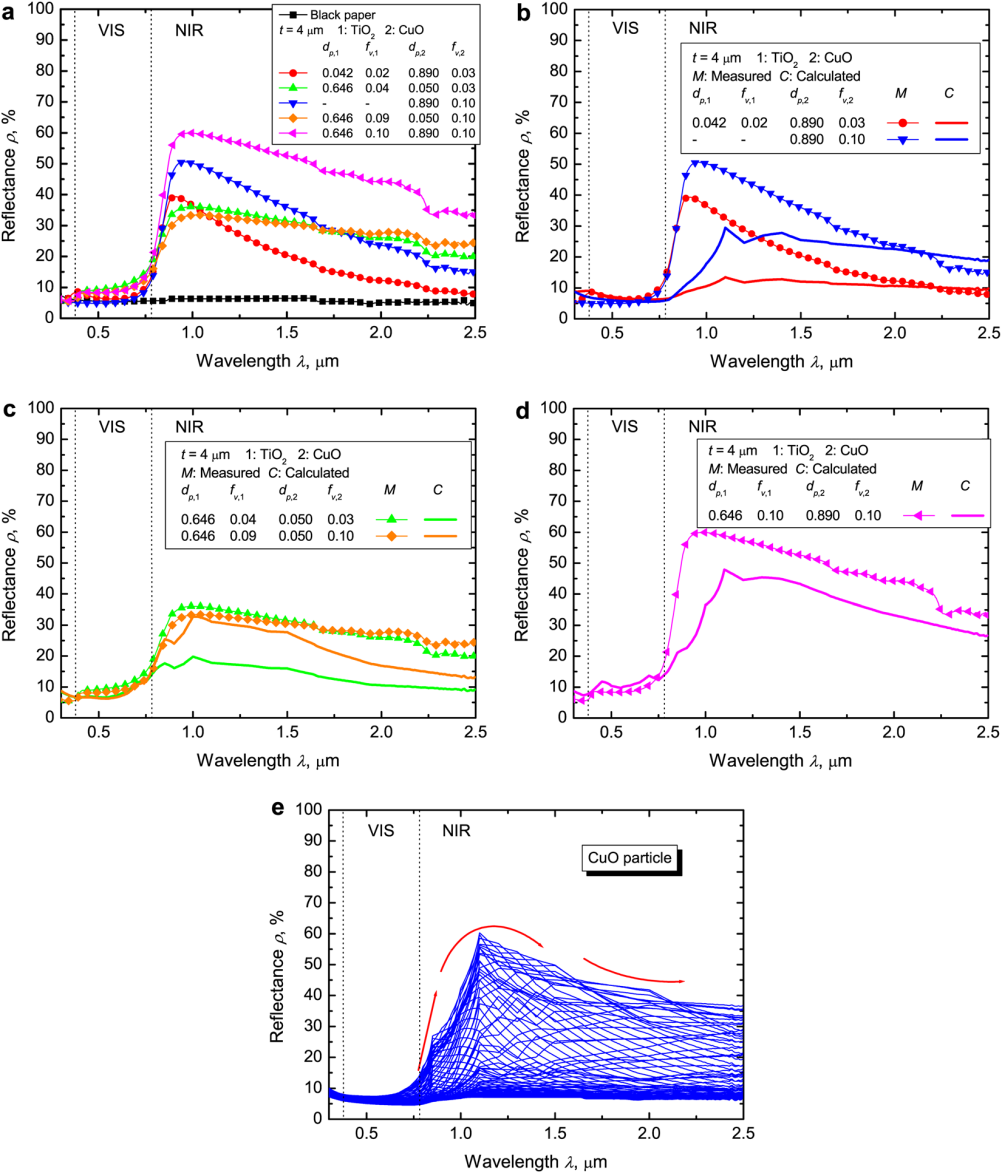
Measured and calculated spectral reflectance of C^3^ when the VIS controlling particle is CuO and the NIR reflecting particle is TiO_2_. (**a**) Measured spectral reflectance of C^3^ when *d*_*p*,1_ is 0.042 μm, *f*_*v*,1_ is 0.02, *d*_*p*,2_ is 0.890 μm, and *f*_*v*,2_ is 0.03 (red curve; *R = *8.18); when *d*_*p*,1_ is 0.646 μm, *f*_*v*,1_ is 0.04, *d*_*p*,2_ is 0.050 μm, and *f*_*v*,2_ is 0.03 (green curve; *R = *6.46); when *d*_*p*,1_ is 0.646 μm, *f*_*v*,1_ is 0.09, *d*_*p*,2_ is 0.050 μm, and *f*_*v*,2_ is 0.10 (orange curve; *R = *6.79); when *d*_*p*,1_ is 0.646 μm, *f*_*v*,1_ is 0.10, *d*_*p*,2_ is 0.890 μm, and *f*_*v*,2_ is 0.10 (pink curve; *R = *10.31); and of the coating pigmented only by a CuO particle when *d*_*p*,2_ is 0.890 μm, *f*_*v*,2_ is 0.10 (blue curve; *R = *12.63), and black paper (black curve). (**b**) Comparison between the measured (point curve) and calculated (bold curve) C^3^ reflectances when *d*_*p*,1_ is 0.042 μm, *f*_*v*,1_ is 0.02, *d*_*p*,2_ is 0.890 μm, and *f*_*v*,2_ is 0.03 (red curve) and the coating pigmented using only the CuO particle when *d*_*p*,2_ is 0.890 μm and *f*_*v*,2_ is 0.10 (blue curve). (**c**) Comparison between the measured (point curve) and calculated (bold curve) C^3^ reflectances when *d*_*p*,1_ is 0.646 μm, *f*_*v*,1_ is 0.04, *d*_*p*,2_ is 0.050 μm, and *f*_*v*,2_ is 0.03 (green curve) and that when *d*_*p*,1_ is 0.646 μm, *f*_*v*,1_ is 0.09, *d*_*p*,2_ is 0.050 μm, and *f*_*v*,2_ is 0.10 (orange curve). (**d**) Comparison between the measured (point curve) and calculated (bold curve) C^3^ reflectances when *f*_*v*,1_ is 0.10, *d*_*p*,2_ is 0.890 μm, and *f*_*v*,2_ is 0.10 (pink curve). (**e**) Calculated reflectance of the CuO pigmented coating at *d*_*p*_ is from 0.010 to10 μm. The spectral tendency shifts with the increment in the particle diameter (red arrow).

We prepared C^3^ and measured its spectral reflectance. We demonstrated that the spectral reflectance can be controlled to be low in the VIS and high in the NIR region by combining two size-controlled particles (Fig. 4a). C^3^ has gray color (a slightly VIS reflectance) when the NIR reflecting particle is TiO_2_ with *d*_*p*,1_ = 0.646 μm (green, orange, and pink curves in Fig. 4a). However, in these cases, C^3^ can maintain a high reflectance in the longer wavelength region from 0.9 to 2.2 μm. On the other hand, C^3^ has an almost black color (slightly VIS reflectance) when the CuO particle simultaneously controls the VIS light and reflects the NIR light (red and blue curves in Fig. 4a). As a consequence, the coating pigmented using only a CuO particle where *d*_*p*,2_ is 0.890 μm and *f*_*v*,2_ is 0.10 has the highest optimization parameter (*R = *12.63). However, C^3^ has the highest spectral reflectance in the NIR region when the TiO_2_ and CuO particles with sizes (*d*_*p*,1_ = 0.646 μm, *d*_*p*,2_ = 0.890 μm) close to the effective particle diameters are combined. Therefore, C^3^ is most effective for passive thermal protection.

### Comparison between the measured and calculated reflectances

To determine the necessary improvements to realize the best possible performance, we compared the experimental and calculated results. In cases where the CuO particle simultaneously controls the low VIS and high NIR reflectances (Fig. 4b), the calculated spectral reflectance was lower than the measured one, and the peak of the calculated spectral reflectance shifted to a longer wavelength region. For the CuO pigmented coating, the peak of the spectral reflectance shifted to a longer wavelength region and increased up to 1.1 μm and decreased from 1.1 μm with an increment in the particle diameter (Fig. 4e). The comparison result indicates that the size of particles in the coating could be smaller than that indicated in the catalog. In other words, the CuO particle size used in the experiment might be much closer to the optimum particle size of *d*_*p*,2_ = 0.572 μm. This condition also affected the difference between the measured and calculated reflectances of C^3^ when the effective diameters of the TiO_2_ and CuO particles (*d*_*p*,1_ = 0.646 μm and *d*_*p*,2_ = 0.890 μm) were combined (Fig. 4d). In cases where the VIS controlling particle is CuO and the NIR reflecting particle is TiO_2_, the calculated values indicate that the reflectance increased with the increment in the volume fraction of the particles (Fig. 4c). However, the measured reflectance of C^3^ at *f*_*v*,1_ = 0.09 and *f*_*v*,2_ = 0.10 was almost the same as that at *f*_*v*,1_ = 0.04 and *f*_*v*,2_ = 0.03. One of these causes appeared to be the coagulations of CuO particles. When the particle size of the CuO particle increased, the extinction efficiency in the NIR region also increased. Small nanoparticles can easily coagulate. Another cause appeared to be the interaction between two different particles. For example, smaller CuO particles are attached to the surface of bigger TiO_2_ particles due to the universal law of gravitation. To achieve a more accurate spectral control, the dependent scattering between two different particles^17–20^ must be considered. We need to more deeply investigate why the difference in the measured and calculated reflectance existed, and thus, we cannot conclude its causes yet.

## Discussions

By combining optical modeling and the experimental method, we develop C^3^ bio-inspired from the passive thermal protection of a chameleon. The superficial iridophores of a chameleon, which controls the VIS light, is imitated using CuO particles to achieve a black color. The deep iridophores of a chameleon, which reflects the NIR light, is imitated using TiO_2_ particles to achieve a radiative thermal barrier. We calculate the spectral reflectance of C^3^ and define the optimization parameter to evaluate the performance of the thermal protection while retaining the color. For optimal spectral reflectance, the balance of the absorption of CuO particles and scattering of TiO_2_ particles is important. Particle size affects the spectrum, and particle volume affects the intensity of absorption/scattering of C^3^.

To improve the thermal-resistance performance, control of the particle size is important. Monodispersed and accurate size-controlled particles yield better thermal-resistance performance, as shown by the theoretical calculation. In particular, the interaction between two different particles affects their dispersed condition; therefore, applying stirring and mixing techniques could improve control of the dispersion/aggregation in a particle cloud. By using the proposed C^3^, the interior temperature decreases and the cooling load can be reduced while keeping the dark tone of the object.

Nature possesses hidden amazing technology. We are inspired by the passive thermal protection of a chameleon. However, many biomimetic challenges in optical engineering need to be addressed. For example, the natural photosynthesis of a plant is mimicked by hollow nano-spheres and used in energy harvesting^21^, a lotus leaf creates a low reflectance black coating^22^, the elytra of longhorn beetles is inspired for color-shifting films, and the blue reflectance of tarantulas inspires future display applications^7^. We expect that more bio-inspired technology will be developed in the future for innovative optical techniques.

## Method

### Radiative properties of a single particle

**Figure 5:**
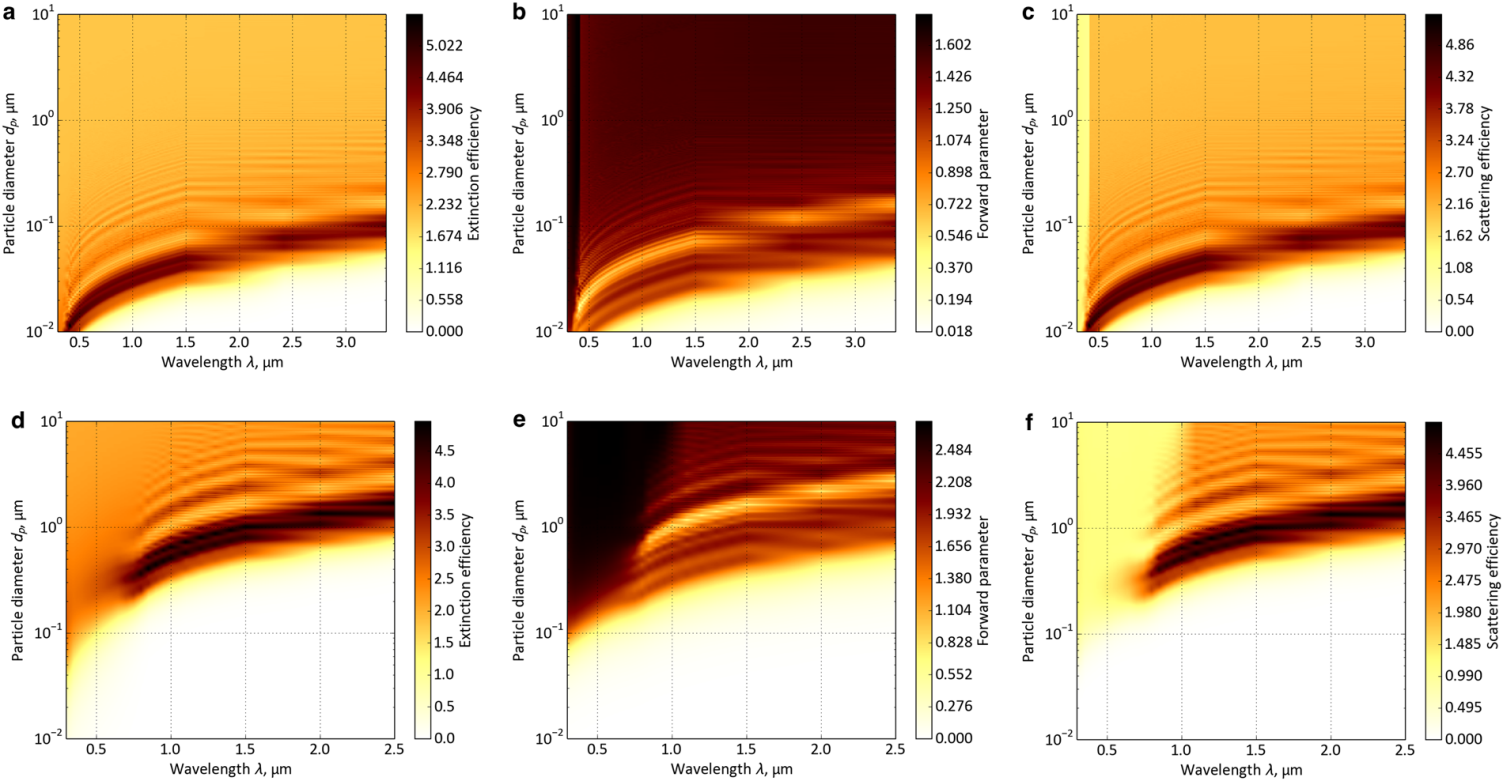
Spectral radiative properties of a single particle as a function of the particle diameter. (**a**) Spectral extinction efficiency of a TiO_2_ particle. (**b**) Spectral forward parameter of a TiO_2_ particle. (**c**) Spectral scattering efficiency of a TiO_2_ particle. (**d**) Spectral extinction efficiency of a CuO particle. (**e**) Spectral forward parameter of a CuO particle. (**f**) Spectral scattering efficiency of a CuO particle.

The scattering and absorption of radiation by a single homogeneous spherical particle in a non-absorbing medium can be obtained by solving Maxwell’s equations. These radiative properties depend on particle diameter *d*_*p*_, wavelength of the incident electromagnetic wave *λ*, and complex refractive index of the particle *m* = *n* − *ik*, as described in the Mie scattering theory^23^. Using the spectral complex refractive indexes of TiO_2_ and CuO^24^, we calculated the radiative properties of single TiO_2_ and CuO particles as a function of the particle size (Fig. 5). The medium was assumed to be a non-absorbing acrylic resin with a refractive index equal to 1.5. From the radiative properties of a single particle, the radiative property of a composite particle cloud was calculated for radiative-transfer analysis.

### Radiative properties of a particle cloud

Assuming an independent scattering, for a group of monodispersed particles, the scattering, extinction, and absorption coefficients can be calculated using the following equations^25^:

1 $${\sigma }_{s,\lambda ,mono}=\pi {(\frac{{d}_{p}}{2})}^{2}{Q}_{sca}{n}_{p}$$

2 $${\beta }_{\lambda ,mono}=\pi {(\frac{{d}_{p}}{2})}^{2}{Q}_{ext}{n}_{p}$$

3 $${\kappa }_{\lambda ,mono}=\pi {(\frac{{d}_{p}}{2})}^{2}{Q}_{abs}{n}_{p}$$

where *σ*_*s*_, *β*, and *κ* are the scattering, extinction, and absorption coefficients, respectively. *n*_*p*_ is the number of particles per unit volume. *Q*_sca_, *Q*_ext_, and *Q*_abs_ are the scattering, extinction, and absorption efficiencies obtained from the Mie calculations^23^, respectively. Because phase function Φ in a cloud of uniform particles is the same in each particle, it is also the same in the particle cloud, i.e.,

4 $${{\rm{\Phi }}}_{mono}(\mu )={\rm{\Phi }}(\mu )$$

Similarly, for forward parameter *a*_1_

5 $${a}_{1,mono}\equiv {a}_{1}$$

where *μ* is the cosine of direction angle *θ*. Moreover, in this case, the total volume of particles per unit volume (or volume fraction) can be calculated using the following equation:

6 $${f}_{v}=\frac{1}{6}\pi {d}_{p}^{3}{n}_{p}$$

In all these equations, subscript “mono” indicates uniform size of particles.

### Radiative properties of a composite particle cloud

Assuming independent scattering, the radiative properties of a composite particle cloud can be expressed by the superposition principle, as expressed in the following equations^26^:

7 $${\sigma }_{mix}={\sigma }_{1}+{\sigma }_{2}$$

8 $${\kappa }_{mix}={\kappa }_{1}+{\kappa }_{2}$$

In all these equations, subscript “mix” indicates the use of two particles made of different materials.

### Radiative transfer analysis

To calculate the radiative transfer in C^3^, C^3^ was treated as a participating medium in a one-dimensional plane parallel system in the analytical model^27^. The radiative transfer in this system can be expressed using the radiative transfer equation (RTE) as follows:

9 $$\frac{d{I}_{\lambda }(x,\mu )}{dS}=\beta [-{I}_{\lambda }(x,\mu )+(1-\omega ){I}_{b,\lambda }(T)+\frac{\omega }{2}{\int }_{-1}^{1}{I}_{\lambda }(x,\mu \text{'}){{\rm{\Phi }}}_{\lambda }(\mu \text{'})d\mu \text{'}]$$

where *I*_*λ*_ is the intensity of the incident radiation, *S* is the path length through an element, *ω* is the albedo, and *I*_*b*_,_*λ*_ is the intensity of the blackbody radiation. To solve the RTE, the radiation element method by ray emission model^28^ was used. Then, apparent scattering coefficient σ^*^, apparent absorption coefficient κ^*^, apparent extinction coefficient β^*^, and corrected albedo *ω*^*D*^ for an anisotropic medium can be expressed as

10 $${\sigma }^{\ast }=\sigma (1-{a}_{1}/3)$$

11 $${\kappa }^{\ast }=\kappa $$

12 $${\beta }^{\ast }=\beta (1-\omega {a}_{1}/3)$$

13 $${\omega }^{D}=\frac{\omega (1-{a}_{1}/3)}{1-\omega {a}_{1}/3}$$

In these calculations, the dispersion state was assumed to be monodispersed to simplify the effective parameter and reduce the computational load^29^. The specular reflection produced by the difference among the refractive indexes was calculated using Fresnel’s equation^25^. The spectral solar irradiation was evaluated using Bird’s model^30^. The substrate was a black paper standardized according to the Japan Industrial Standards whose spectral reflectance was measured. The scattering was assumed to be independent.

### Definition of the optimization parameter

The optimization parameter was defined to determine a suitable pigment particle^27^. The performance parameter of the pigmented coating for solar reflectance in the solar spectrum region is expressed as

14 $${\rho }_{TSR}=\frac{{\int }_{0.30}^{2.50}\rho (\lambda )I(\lambda )d\lambda }{{\int }_{0.30}^{2.50}I(\lambda )d\lambda }$$

where *I* is the solar irradiation and *ρ* is the spectral reflectance of the pigmented coating. A parameter that evaluates the aesthetic performance of a pigmented coating by considering the spectral eye sensitivity is defined as follows:

15 $${\rho }_{VIS}=\frac{{\int }_{0.38}^{0.78}\rho (\lambda )\eta (\lambda )I(\lambda )d\lambda }{{\int }_{0.38}^{0.78}I(\lambda )d\lambda }$$

where *η* is the normalized standard luminous efficiency. Optimization parameter *R* can be calculated as follows:

16 $$R=\frac{{\rho }_{TSR}}{{\rho }_{VIS}}$$

To achieve an optimized coating, *R* should be maximized.

### Preparation of C^3^

For the experiment, C^3^ was prepared using a clear acrylic synthetic resin as the matrix and a standard black paper as the substrate. Spherical CuO particles were used as VIS-control particles, which have nominal mean particle diameters of 0.050 μm (544868, Sigma-Aldrich Co. LLC) and 0.890 μm (CUO12PB, Kojundo Chemical Laboratory Co., LTD). Spherical TiO_2_ particles were used as NIR reflecting particles, which have nominal mean particle diameters 0.042 μm (MT-500B, Tayca Corporation) and 0.890 μm (JR-1000, Tayca Corporation). Purities of all particles are over 99% without surface treatment. The nominal mean diameters and impurity data were provided by the respective chemical supplier companies.

First, the particles were weighted using a balance (AE 163, Mettler). Then, the particle volume fraction C^3^ could be evaluated from the weight of the particles using the following equation:

17 $${f}_{v,1}=\frac{({m}_{1}/{\rho }_{1})}{({m}_{1}/{\rho }_{1})+({m}_{2}/{\rho }_{2})+V},\,{f}_{v,2}=\frac{({m}_{2}/{\rho }_{2})}{({m}_{1}/{\rho }_{1})+({m}_{2}/{\rho }_{2})+V}$$

where *f*_*v*_ is the particle volume fraction, *m* is the weight of the particles, *ρ* is the density of the particle material, and *V* is the volume of the acrylic synthetic resin. To avoid a significant error in our results due to particle coagulation and crack in the coating, the volume fractions were configured up to 0.10. To break the coagulation of particles, thinner (MR. COLOR, GSI Creos Corporation) was mixed, and an ultrasonic device (USM-1, AS ONE) was used for 5 min. The mixture was mixed with the acrylic resin (MR. COLOR, GSI Creos Corporation) using a super mixer (AR-100, THINKY). The mixing time was set at 5 min for stirring and 1 min for defoaming. The mixture was dispersed over the substrate using a spiral bar coater (Elcometer). After coating, the samples were dried in air. The thickness of C^3^ was measured using a digital micrometer.

### Spectral-reflectance measurement

The measurement of the spectral reflectance in the solar wavelength range of 0.3–2.5 µm was performed using ultraviolet–VIS–NIR spectrophotometers (UV-3600, Shimadzu) and an integrating sphere (MPC-603, Shimadzu). The measured reflectance was calibrated using standard reflecting plate Spectralon (SRS-99-020, Labsphere).

## References

[1] Siddique RH, Vignolini S, Bartels C, Wacker I, Hölscher H (2016). Colour formation on the wings of the butterfly Hypolimnas salmacis by scale stacking. Scientific Reports.

[2] Yoshioka S, Kinoshita S (2006). Structural or pigmentary? Origin of the distinctive white stripe on the blue wing of a Morpho butterfly. Proceedings of the Royal Society B: Biological Sciences.

[3] Banerjee S, Cole JB, Yatagai T (2007). Colour characterization of a Morpho butterfly wing-scale using a high accuracy nonstandard finite-difference time-domain method. Micron.

[4] Teyssier, J., Saenko, S. V., van der Marel, D. & Milinkovitch, M. C. Photonic crystals cause active colour change in chameleons. *Nat Commun* **6** (2015). 10.1038/ncomms7368 PMC4366488 25757068

[5] Yu K, Fan T, Lou S, Zhang D (2013). Biomimetic optical materials: Integration of nature’s design for manipulation of light. Progress in Materials Science.

[6] Xu J, Guo Z (2013). Biomimetic photonic materials with tunable structural colors. Journal of Colloid and Interface Science.

[7] Hsiung, B.-K., Deheyn, D. D., Shawkey, M. D. & Blackledge, T. A. Blue reflectance in tarantulas is evolutionarily conserved despite nanostructural diversity. *Science Advances* **1** (2015). 10.1126/sciadv.1500709 PMC4681340 26702433

[8] Martín-Palma RJ, Lakhtakia A (2017). Progress on bioinspired, biomimetic, and bioreplication routes to harvest solar energy. Applied Physics Reviews.

[9] Chattopadhyay S (2010). Anti-reflecting and photonic nanostructures. Materials Science and Engineering: R: Reports.

[10] Li Y, Zhang J, Yang B (2010). Antireflective surfaces based on biomimetic nanopillared arrays. Nano Today.

[11] Levinson R (2010). A novel technique for the production of cool colored concrete tile and asphalt shingle roofing products. Solar Energy Materials and Solar Cells.

[12] Suehrcke H, Peterson EL, Selby N (2008). Effect of roof solar reflectance on the building heat gain in a hot climate. Energy and Buildings.

[13] Synnefa A, Santamouris M, Apostolakis K (2007). On the development, optical properties and thermal performance of cool colored coatings for the urban environment. Solar Energy.

[14] Baneshi M, Gonome H, Maruyama S (2016). Cool black roof impacts into the cooling and heating load demand of a residential building in various climates. Solar Energy Materials and Solar Cells.

[15] Seneviratne SI, Donat MG, Pitman AJ, Knutti R, Wilby RL. Allowable CO2 emissions based on regional and impact-related climate targets. *Nature***advanced online publication** (2016). 10.1038/nature16542 26789252

[16] Saitoh TS, Yamada N (2004). Experimental and numerical investigation of thermal plume in urban surface layer. Experimental Thermal and Fluid Science.

[17] El-Sayed MA (2004). Small is different:  Shape-, size-, and composition-dependent properties of some colloidal semiconductor nanocrystals. Accounts of Chemical Research.

[18] Koya AN, Lin J (2017). Charge transfer plasmons: Recent theoretical and experimental developments. Applied Physics Reviews.

[19] Singh BP, Kaviany M (1992). Modelling radiative heat transfer in packed beds. International Journal of Heat and Mass Transfer.

[20] Noguez C (2007). Surface plasmons on metal nanoparticles:  The influence of shape and physical environment. The Journal of Physical Chemistry C.

[21] Sun J (2012). Bioinspired hollow semiconductor nanospheres as photosynthetic nanoparticles. Nat Commun.

[22] Ebihara Y, Ota R, Noriki T, Shimojo M, Kajikawa K (2015). Biometamaterials: Black ultrathin gold film fabricated on lotus leaf. Scientific Reports.

[23] Mie G (1908). Beiträge zur Optik trüber Medien, speziell kolloidaler Metallösungen. Annalen der Physik.

[24] Palik E. D. *Handbook of Optical Constants of Solids*. Academic Press (1998).

[25] Modest M. F. *Radiative Heat Transfer*. Academic Press (2003).

[26] Gonome H, Ishikawa Y, Kono T, Yamada J (2017). Radiative transfer analysis of the effect of ink dot area on color phase in inkjet printing. Journal of Quantitative Spectroscopy and Radiative Transfer.

[27] Gonome H, Baneshi M, Okajima J, Komiya A, Maruyama S (2014). Controlling the radiative properties of cool black-color coatings pigmented with CuO submicron particles. Journal of Quantitative Spectroscopy and Radiative Transfer.

[28] Maruyama S, Aihara T (1997). Radiation heat transfer of arbitrary three-dimensional absorbing, emitting and scattering media and specular and diffuse surfaces. Journal of Heat Transfer.

[29] Baneshi M, Gonome H, Komiya A, Maruyama S (2012). The effect of particles size distribution on aesthetic and thermal performances of polydisperse TiO_2_ pigmented coatings: Comparison between numerical and experimental results. Journal of Quantitative Spectroscopy and Radiative Transfer.

[30] Bird RE, Riordan C (1986). Simple solar spectral model for direct and diffuse irradiance on horizontal and tilted planes at the Earth’s surface for cloudless atmospheres. Journal of Climate and Applied Meteorology.

